# Integration of systematic screening for tuberculosis in outpatient departments of urban primary healthcare facilities in Zambia: a case study of Kitwe district

**DOI:** 10.1186/s12913-022-08043-w

**Published:** 2022-06-02

**Authors:** Davy Wadula Zulu, Adam Silumbwe, Patricia Maritim, Joseph Mumba Zulu

**Affiliations:** 1 Department of Health Policy and Management, School of Public Health, University of Zambia, Lusaka, Zambia; 2 Department of Epidemiology and Global Health, Umeå University, 901 87 Umeå, Sweden

**Keywords:** Facilities, Implementation, Integration, Primary healthcare, Systematic screening, Tuberculosis

## Abstract

**Background:**

Tuberculosis (TB) is the leading cause of death from a single infectious agent globally, killing about 1.5 million people annually, yet 3 million cases are missed every year. The World Health Organization recommends systematic screening of suspected active TB patients among those visiting the healthcare facilities. While many countries have scaled-up systematic screening of TB, there has been limited assessment of the extent of its integration into the health system. This study sought to explore factors that shape the integration of systematic screening of TB in outpatient departments of primary healthcare facilities in Kitwe district, Zambia.

**Methods:**

This was a qualitative case study with health providers including district managers, TB focal point persons and laboratory personnel working in six purposively selected primary healthcare facilities. Data was collected through key informant (*n* = 8) and in-depth (*n* = 15) interviews. Data analysis was conducted using QDA Miner software and guided by Atun’s Integration framework.

**Results:**

The facilitators to integration of systematic screening for TB into out patient departments of primary health facilities included the perceived high burden TB, compatibility of the systematic screening for TB program with healthcare workers training and working schedules, stakeholder knowledge of each others interest and values, regular performance management and integrated outreach of TB screening services. Constraining factors to integration of systematic screening for TB into outpatient departments included complexity of screening for TB in children, unbalanced incentivization mechanisms, ownership and legitimacy of the TB screening program, negative health worker attitudes, social cultural misconceptions of TB and societal stigma as well as the COVID-19 pandemic.

**Conclusion:**

Systematic screening of TB is not fully integrated into the primary healthcare facilities in Zambia to capture all those suspected with active TB that make contact with the health system. Finding the missing TB cases will, therefore, require contextual adaptation of the systematic screening for TB program to local needs and capacities as well as strengthening the health system.

**Supplementary Information:**

The online version contains supplementary material available at 10.1186/s12913-022-08043-w.

## Background

Globally, tuberculosis (TB) remains one of the leading cause of death from a single infectious agent, killing about 1.5 million people each year [1, 2]. It is estimated that 85% -95% of these deaths could be prevented with early detection, diagnosis and appropriate treatment. Yet more than 3 million TB cases are missed (either not diagnosed or diagnosed but not notified) annually [1]. Thus, the importance of promptly identifying TB cases and linking them to care cannot be over emphasized.

In the past 25 years, global TB control has seen the adoption of three main strategies. The Direct Observed Treatment (DOTS) strategy of 1994 to 2005 emphasized passive case finding [3]. This was followed by the STOP TB strategy of 2006 to 2015 which emphasized intensified case finding in health facilities, communities and congregate settings [4]. Currently, the END TB strategy since 2016 proposes active case finding and contact tracing among populations at risk [5]. Through these strategies, 58 million TB deaths were averted between the years 2000 and 2018 [6]. However, there are fundamental gaps hampering the success of these global strategies, particularly in resource-constrained settings [7].

Evidence suggests that the missed TB cases are not really missing as most of them are actively engaging with the health system which is failing to appropriately capture them [8]. This is due to various factors including low TB screening and testing capacity, poor understanding of the screening protocols, inadequate knowledge by the health providers to suspect TB, low diagnostic capacities and shortages of inputs[9–11]. Further, the growing interest in community-based active case finding (ACF) strategies especially in resource-constrained settings has shifted the attention from [12, 13] facility-based intensified TB case finding even though it is more cost-effective and efficient [14, 15].

The World Health Organization (WHO) underscores missed TB cases as a significant contributor to continued high prevalence and incidence of TB in low- and middle-income countries (LMIC) and recommends systematic screening of all suspected active TB patients who come to the health facility [6]. Systematic screening for TB is the systematic identification of people with suspected active TB, in a predetermined target group, using tests, examinations or other rapid procedures [16]. While many LMICs have scaled-up systematic screening of TB, there has been limited assessment of the extent of its integration into the health system [1].

**Fig. 1:**
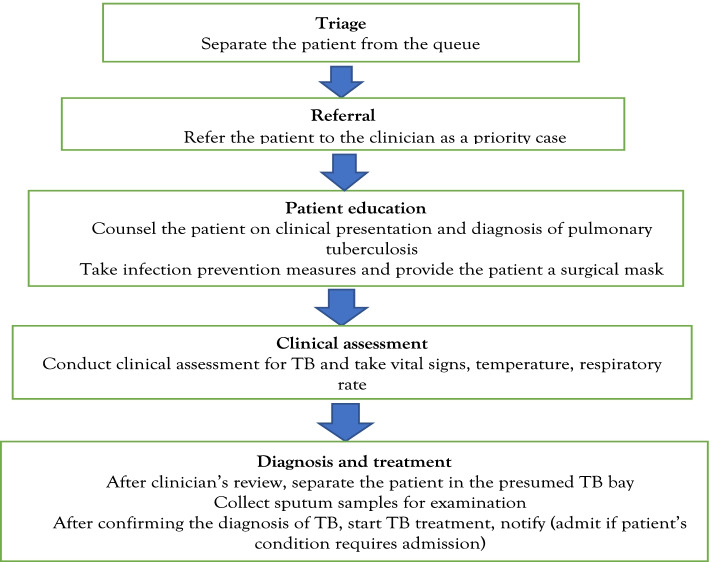
Phases of systematic screening for TB in out-patient departments

Zambia is one of the top 30 high TB burden countries with an estimated incidence of 346/100,000 population compared to a global average of 130/100,000 [17]. Disease control efforts in Zambia are guided by the National Strategic Plan for tuberculosis prevention, care and control 2017–2021 (NSP) and the national TB manual of 2017. Both policy documents outline systematic screening for TB at health facility level as a key strategy for detection of TB cases [18, 19]. This is done through symptomatic screening for cough, blood in sputum, chest pains, fever, night sweats and weight loss (Fig. 1).

Despite the implementation and roll out of systematic screening for TB over the past 10 years in Zambia, about 50% of symptomatic TB patients who seek healthcare from various health facilities are not investigated for TB [20]. This could be due to poor integration of the systematic screening for TB into the health system. Optimal integration of TB screening interventions into the health systems has been shown to improve patient care outcomes and produce close to desired results [21]. However, few studies have been done in Zambia to understand how systematic screening for TB is integrated into primary healthcare systems. This study, therefore, sought to explore factors that shape the integration of the systematic screening for TB in the outpatient departments (OPDs) of primary health facilities with a view of finding contextualized solutions to improve TB case detection and treatment. We define integration as the extent, pattern, and rate of adoption and eventual assimilation of systematic screening for TB into each of the critical functions of a health system [22].

## Methods

### Research design

A qualitative case study design was employed as it is appropriate for exploring integration in the context of an urban primary healthcare facilities of Kitwe district [23]. This design allowed us to uncover context specific implementation issues affecting integration of systematic screening in primary healthcare facilities. We used the consolidated criteria for reporting qualitative research (COREQ) to guide the research process (additional file).

### Study setting

Kitwe district is located in the central part of the Copperbelt Province of Zambia. It is the second most populated district in Zambia after the capital, Lusaka with about 66 percent of the population below the age of 25 years [24]. The main economic activities in the district are mining, agriculture and tourism. At the time of study, the district delivered its healthcare services through 3 hospitals and 39 primary healthcare facilities coordinated by the District Health Office. All 39 primary health facilities had instituted TB screening in OPD and additional settings but only 12 had TB diagnostic capabilities. Therefore, the facilities without diagnostic capacity referred sputum samples or patients to these diagnostic health facilities. Kitwe district was chosen as a case because it recorded a high TB incidence and the high number of TB patients that interacted with the health facilities but were not investigated [20].

### Participant recruitment and sampling approach

All primary health care facilities in Kitwe District were listed and six were purposively selected with maximum variation in TB notifications (three facilities with the highest and the other three with lowest TB notifications). The final selection of health facilities was subject to consent from the health facility in-charges. District TB managers working in the selected district were purposively selected by the research team and helped to contact facility in-charges who in turn helped in the identification of OPD clinicians, laboratory personnel (where available), and health facility TB focal point persons. District TB managers were chosen because they provide preside over the TB programs including the systematic screening for TB while the HCWs were selected because they are implementers of systematic screening for TB in the health facilities. The sample size was determined by the principle of theoretical saturation [25]. Saturation is the point at which no new data can be obtained from the interviews and thus further data collection is unnecessary [26]. Though we planned to conduct twenty-six [25] interviews, saturation was reached after 20 interviews but three more interviews were added to be certain that the saturation point was reached.

### Data collection

Data were collected between November 2020 and January 2021 through a total of twenty-three (*n* = 23) face-to-face key informant (KIIs) and in-depth interviews (IDIs) (Table 1). Interview guides developed according to the objectives of the study and the theoretical framework were used for data collection. All interviews were conducted in English and lasted between forty-five and eighty minutes. The interviews were digitally recorded with consent from participants and field notes were also taken. The KIIs for the district TB managers were conducted at the district health office and all the other interviews were conducted at the respective health facilities observing privacy and COVID-19 prevention measures. Data were transcribed verbatim. During and at the end of the interviews, a summary of the responses was repeated to each participant to confirm if their responses were captured and interpreted correctly.

**Table 1:** IDI and KII participants

Data collection method/participants	Number of interviews
**Key informant interviews**	
District TB program managers	2
Health facility in-charges	6
**Total KIIs**	**8**
**In-depth interviews**	
Laboratory Personnel	5
TB Focal Point Person	4
OPD Clinician	6
**Total IDIs**	**15**
**Total Interviews**	**23**

### Data analysis

A thematic analysis approach to explore relationships and patterns in qualitative data was used [27]. The data analysis process started during data collection by DWZ in order to learn and refine the interview questions. It was an on-going process that involved arranging the field notes according to themes in relation to the objectives and the framework. DWZ listened to participants’ recorded responses several times for familiarization. Field notes and comments were regularly reviewed in line with the themes and a list of major ideas that surfaced were added to a code list which was later imported into QDA Miner software. Six transcripts were shared among AS, PM and JMZ to verify the content and consolidate the coding. Subsequent iterative discussions among the research team allowed for additional modifications of the coding structure. Inductive and deductive coding approaches were used to ensure both framework-guided and emergent subthemes in the data were captured. Data was triangulated across different categories of participants and health facilities.

**Fig. 2:**
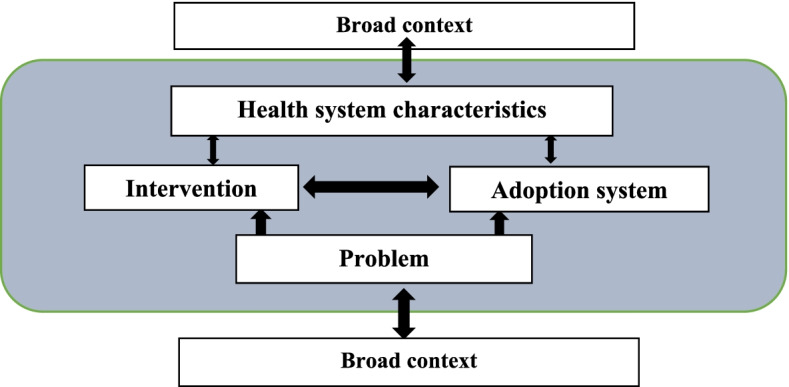
Atun’s integration framework

The data collection, analysis and reporting of findings was guided by Atun’s integration framework which posits that the integration of health interventions into health systems is influenced by; the nature of the problem being addressed, the intervention, the adoption system, the health system characteristics, and the broader context [28]. From this, we theorized that the nature of the TB problem such as its burden may influence actors’ perspectives towards TB guidelines thus shaping the integration process. Attributes of the systematic screening for TB such as the complexity and compatibility of its components with the work culture and values of the actors in the adopting system–including program managers, health workers and the health system characteristics such as resources, capacity building, demand creation, performance and regulatory systems may also influence the integration process. Factors in the broader context such as the demographic, economic, political and socio-cultural factors may shape integration of the systematic screening for TB into the health system by affecting any of the above factors (Fig. 2).

### Ethical approval and consent to participate

Ethical clearance was sought from the University of Zambia Biomedical Research Ethics Committee (REF. 1015–2020). Permission was also sought from the National Health Research Authority (NHRA), the Ministry of Health as well as Kitwe District authorities to conduct the study. Both signed and verbal informed consent were obtained from the participants. COVID-19 preventive measures were adhered to through provision of face masks, hand sanitizer and social distancing during data collection. All the participants were de-identified to guarantee confidentiality. All methods were carried out in accordance with relevant guidelines and regulations.

## Results

We present our findings on the factors that shape the integration of systematic screening into the OPDs of primary healthcare facilities. The themes have been organized around the constructs of Atun’s framework on integration of health interventions into health systems, namely: characteristics of the problem, attributes of the intervention, the adoption system, the health system characteristics and the broader context (Table 2) [28]. Although data were collected from various participant categories, no major differences in the discussions were noted and views specific to a particular participant category are noted within the manuscript.

**Table 2:** Key thematic categories

Broad themes	Sub-theme
The nature of the TB problem	▪ Perception of the TB burden
The intervention	▪ Alignment with HCWs skills, training and work schedules
	▪ Complexity in screening children
The adoption system	▪ Stakeholders’ knowledge of each other’s interests, values and power
	▪ Incentivization mechanisms
	▪ Influence from external partners
Health system characteristics	▪ Consistent and decentralized performance management
	▪ Continuous capacity building
	▪ Integrated outreach TB screening services
	▪ Negative health worker attitudes
Broad context of integration	▪ Political will
	▪ Socio-cultural misconceptions and gender norms
	▪ The COVID-19 pandemic

### Factors that shape the integration of systematic screening for Tuberculosis in OPDs of primary healthcare facilities

#### Nature of the TB problem

##### Perception of the TB burden

It was interesting to find different perceptions of the TB burden among HCWs in the same health facilities. Most clinicians perceived the TB burden to be high and considered it to be a serious public health threat due to the high numbers of TB patients seen in the OPDs. The surrounding environment where majority of TB patients originated was said to play a huge role in driving the TB burden because most of it was low socioeconomic housing. However, some facility in-charges thought that the TB burden was lessening because of the community TB screening programs that had been implemented in the recent past. The perception of a high TB burden increased the index of suspicion for TB which facilitated integration as stated by a clinician:

*“Looking at …the environment of this community and the number of [TB] cases that I see, I would say the burden is high and so I make sure I do my best...to catch them.” Clinician-02, LP*

On the contrary, the facility in-charge from the same facility argued that:

*“As for TB, I can say not so much. Why? Because we don't have a lot of TB patients due to various community screening programs conducted in the past.” IC-02, LP*

#### The intervention

##### Alignment with HCWs’ training, skills and work schedules

Most HCWs agreed that the principles and concepts of the systematic screening for TB were relatively easy to understand and apply. They were within their in-service training and skills, making it easy for them to adopt. The compatibility of systematic screening for TB with clinicians’ and laboratory staff’s routine work schedule facilitated the integration process as they did not have to change the flow of patients.

*“…our training is enough. Our work is not disturbed…We just need to keep in mind that we need to ask about TB in all patients who come through.” Clinician-06, LP*

However, the intervention was reported not to be compatible with the pre-service training and work schedules of the appointed facility TB focal point persons who were all nurses. They had to adapt by learning new concepts such as TB drugs and their side effects, treatment algorithms, follow-up of patients, reporting systems and templates. Those who could not adapt well were thus unable to integrate the intervention into the OPDs. Moreover, some of them were still attached to and working in their routine departments thus having a divided work schedule which affected integration of TB systematic screening into the OPD.

*“I am a nurse but now I need to work like a clinical officer, I had to learn the drugs and how to treat a TB patient…through mentorship. I also write all the TB reports for the facility. I never thought I could change like this…” TB-FPP-01, HP*

##### Complexity in screening of children

All the clinicians noted that it was difficult to integrate systematic TB screening for children in the OPDs because children presented with atypical symptoms which were not included in the screening tools. In addition, children with suspected TB cases were unable to expectorate sputum and it was time-consuming and difficult to perform a gastric lavage. The additional time required to conduct the systematic screening for TB effectively created pressure of work, especially in high patient volume healthcare facilities, which was a barrier to integration.

*“With the adults, it's a straightforward thing, but with the children, there are hiccups because they are presenting not with all the features of TB on the screening tools and lavage is time-consuming, difficult and uncomfortable.” Clinician-05, HP.*

#### The adopting system

##### Stakeholders’ knowledge of each other’s interests, values and power

Stakeholders’ knowledge of each other’s interests, values and power facilitated integration of the systematic screening for TB in the OPDs. For instance, the district team’s knowledge of the funders’ financial power and interest to achieve project targets and improve the district TB indicators encouraged them to ensure HCWs sustain TB screening and notification.

*“Our partners want results…we are making sure that all our TB programs are done and the facilities are doing what they are supposed to do as far as TB screening and notifications are concerned.” Manager-01*

Similarly, HCWs aligned their activities to the district and partners’ values and interests to meet the TB targets set for them in order to receive more funding and recognition. By so doing they increased the extent of assimilation of the systematic screening for TB into the OPDs while meeting the community expectations of satisfactory TB services.

*“Someone [a CBV] goes out …will collect any sputum… just to meet the quarterly target and get paid but that defeats science. So, we put more incentives for a positive TB result…for them to do it right always.” Lab-02, HP*

##### ***Incentivization mechanisms***

Most clinicians noted that there was an imbalance in the incentives among the implementers of the intervention because the TB focal point persons in the health facilities enjoyed better incentives through workshops and trainings. Further, the TB focal point persons received recognition from the district team and funders for the targets achieved than the clinicians. This difference in incentives demotivated clinicians from routinely screening for TB and hindered sustainability of the internvetion.

*“…you see, these guys, the focal persons when they see that this workshop involves money, they will go, yet they don't screen. Instead of sending the actual people who screen the patients. …It is very demotivating...” Clinician-05, HP*

.

##### Ownership and legitimacy of the TB program

Most facility level participants sited the ever-increasing influence of external partners in what TB activities are implemented, when and who is involved as a hinderance to integration of systematic screening in OPDs. HCWs’ lack of involvement at the planning stage coupled with prioritization of funders’ activities made them question the ownership of the TB screening program as well as the legitimacy of TB screening targets set by the district and cooperating partners. The dependence on donor-funding for most of the TB programs including training of HCWs and TB screening activities seemed to validate the HCW's concerns regarding ownership. This compromised the integration of systematic screening for TB into routine OPD services.

*“Sometimes we have programs but partners come in with other programs... They even come with targets that we do not know where they came from…” IC-03, LP*

#### Characteristics of the health system

##### Consistent and decentralized performance management

HCWs agreed that regular monitoring and supervision by the district team motivated them to consistently screen for TB in the OPD. Decentralizing this performance management to the health facilities through facility in-charges and heads of departments within the primary healthcare facilities was effective in ensuring sustained TB screening. The decentralized supervision further fostered the adoption of systematic screening for TB into all entry points of the health facility. Regular data reviews at facility level enabled quick adaptive measures and integration of TB screening into the OPDs.

*“Before the district come...I am the center of the supervision for screening; but in each department, we have heads. So, with the help of those heads in those departments, we are the ones who are doing it... every head of the Department has been given the responsibility to ensure that such activities [TB screening] are going on every day.” TB-FPP-1, HP.*

##### Continuous capacity building

Continuous capacity building through on-site mentorship of HCWs helped to maintain a high index of suspicion for TB and ensured compatibility of the intervention with the OPD. The competences built in the HCWs made them confident to accept and adopt the systematic screening for TB in their routine work. Onsite mentorship within the facility was considered to be a good initiative that fostered integration because it involved all HCWs and brought out contextualized solutions to the challenges they faced in the delivery of systematic screening services for TB.

*“…So, the onsite membership, for me, it has really helped us. … it makes me confident to do it [integrate systematic screening for TB into routine work]. They [the district mentorship team] keep on coming for mentorship and technical support… to maintain high suspicion index for TB…” IC-01, HP*

##### Integrated outreach of TB screening services

All participants noted that combining mobile TB screening services in the community with under-5 clinics and other periodic programs like child health week increased its acceptance in the health facilities which facilitated integration. Some TB focal point persons and facility in-charges further added that the use of community based volunteers (CBVs) and the involvement of traditional healers, who are trusted members of society, in referring suspected TB patients increased demand systematic screening of TB.

*“So, when you use community members [CBVs] in outreach TB screening and education while doing other programs like under-5 clinics and child health week…It will be very easy for them to accept even when they come to the facility.” IC-03, HP*

##### Negative health worker attitudes

Facility in-charges lamented the lack of interest and poor attitude towards systematic screening for TB among some HCWs in the OPDs who only screened when there was someone monitoring them. This lack of interest in TB screening by the HCWs compromised theconsistent application of the intervention and thus was a barrier to its integration in the OPDs. Further, the lack of interest bred a negative attitude towards patients making it hard for patients to accept TB screening.

*“Some have no interest in screening for TB…because we may know how to screen quite alright but …if we don’t care how we treat them [patients]… can they accept it? …I think the main reason is just the attitude.” IC-04, LP*

#### The broader context

##### ***Political will***

District managers were encouraged by the political will from the government to ensure that all facilities had infrastructure, HCWs, commodities and equipment to provide TB screening services despite financial constraints. They stated that the provision of these supplies in the facilities would increase the integration of systematic screening for TB into the OPDs because the testing would be quicker and of good quality.

*“…when you look at equipment and staff, I think we have been given. I think we have more than fifteen or so laboratories which are actually doing screening using the GeneXpert for instance, it has helped us to do tests faster and I think with quality... We may need more but I am saying… I think the government is doing quite well.” Manager-01*

##### Socio-cultural misconceptions and gender norms

Most participants confirmed the persistent presence and negative effects of socio-cultural misconceptions about TB. Beliefs that TB was contracted by sleeping with a woman during their menses or by being bewitched reduced the acceptability of systematic screening. The association of TB with HIV also propagated stigma which hindered people from screening for TB for fear of societal rejection and humiliation. Some TB focal point persons and facility in-charges noted that women were more affected by TB-associated stigma. Most women would refuse to test for TB due to the possible consequences they would face from their partners such as shaming, beating and even divorce if they were found positive.

*“…So even when you find them with TB, they wouldn't want to disclose to the partners because they are …scared that those partners may chase them away or divorce, or things like that. … some would …totally refuse to test.” IC-05, HP*

##### ***The COVID-19 pandemic***

All participants agreed that during the peak period of COVID-19, OPD attendances, health education sessions and duration of clinical assessment reduced for fear of contracting the disease. The guideline not to wear masks while processing TB samples conflicted with the COVID-19 guidelines to were masks at all times making laboratory personnel reluctant to process TB samples. Further, there was a shift in clinician concentration to COVID-19 which compromised the integration of systematic screening for TB into the OPDs.

*“…COVID has caused a lot of challenges … the concentration has slightly shifted…from these other causes… Instead …you tend to screen faster so that the patient leaves your room {laughing}.” Clinician-05, HP*

## Discussion

This study explored factors that shape the integration of systematic screening for TB into the OPDs of primary healthcare facilities in order to improve the detection, notification and treatment. Facilitators of integration included perceived high burden of TB, alignment of systematic screening for TB with HCWs’ training and work schedules, stakeholders’ knowledge of each other’s interests and values, consistent performance management, and integrated outreach TB screening services in a broader context of political will. Barriers to integration included complexity in screening of children, unbalanced incentivization mechanisms, ownership and legitimacy of the systematic screening for TB program, negative staff attitudes, socio-cultural misconceptions and societal stigma as well as the COVID-19 pandemic.

HCWs’ perception of a high burden of TB in their respective catchment areas was associated with a high suspicion index for TB which facilitated integration of systematic screening for TB into the OPDs. There is evidence that TB cases are missed due to lack of clinical suspicion [29–31] and that a high index of suspicion is associated with higher TB screening and detection rates [32, 33]. Given the importance of perception of the problem on the index of suspicion, a deliberate effort must be made by policy makers to engage frontline HCWs to raise awareness of the magnitude of TB for successful, integrated and sustained implementation.

Unsurprisingly, the difficulty to screen for TB in children hindered integration of systematic screening into the OPDs. This is confirmed by other studies which found that HCWs were reluctant to screen for TB in children due to children’s nonspecific clinical presentation, inability to expectorate sputum and the difficulty of aspirating a sputum sample [34–40]. Apart from hindering integration, this systematic leaving out of children might create a health system propagated inequity in implementation which could be mitigated by increasing the numbers and capacities of HCWs. In addition, integration of systematic screening for TB can be enhanced by aligning it with the HCWstraining and work schedules. Similarly, a systematic review of staff-reported barriers and facilitators to implementation of hospital-based interventions showed that suitability of an intervention to HCWs influenced how easily it was integrated [41].

 Similar to other studies [42–45], stakeholders’ knowledge of each other’s interests and values enhanced collaboration which facilitated integration of systematic screening for TB in OPDs. However, the unintended effects of unbalanced stakeholder incentives, HCWs lack of ownership of the TB program and questions on the legitimacy of TB screening targets demotivated HCWs thus constraining integration of systematic screening for TB in OPDs [46]. Policy makers must engage stakeholders at all stages of the systematic screening process, outlining the purpose, context, rolesand funding in order to enhance its integration in OPDs.

Decentralized performance management and continuous capacity building of HCWs through regular on-site mentorship enabled contextualized solutions hence facilitating integration of systematic screening for TB in OPDs. The preference of on-site mentorship by HCWs [47] implies that it should be prioritized by national TB programs and various stakeholders to sustain integration of systematic TB screening in OPDs. However, the deependance of these activities on international partners undermines local ownership thus constraining integration [48]. Further, negative HCWs' attitudes constrain integration of systematic screening for TB in OPDs [49]. TB programs, including systematic screening should consider addressing behavioral factors of the implementers to enhance their integration in routine practice.

Therole of political will in the integration of interventions in health systems as part of the broader context is supported by studies, on community-based health workers and cervical cancer programs [50–53]. However, societalstigma perpetuated by the association of TB with HIV discouraged patients, especially women, from being screened for fear of discrimination and rejection. Similar results have been reported in a national-wide survey on TB-associated stigma in Ethiopia [54]. All interventions designed to increase integration of systematic screening for TB into health systems must address stigma and its interaction with gender [55]. Furthermore, the fear of, and shift of clinical attention to COVID-19 increased the likelihood of missing TB cases at the health facilities [56]. Public health emergencies such as the COVID-19 pandemic have an overall impact on the broader systems context which negatively affected integration of systematic screening for TB into primary health systems.

### Study limitations and strengths

One limitation of the study is that the sample from Kitwe District may not represent views of other primary health facilities in Zambia, but still provides critical information and learning points on the integration of systematic screening for TB into the primary health system. In addition, the perceptions of healthcare workers and their supervisors are only one part of the picture, and their views needed to be complemented with views of community members. However, the rich description of phenomena, our triangulation of data and adherence to principles of trustworthiness all helped in developing a credible and dependable account of what we believe provides a valuable contribution to the knowledge base on factors that shape the integration of systematic screening of TB into the primary health system.

## Conclusion

Integration of systematic screening for TB into the primary healthcare system can improve indicators of TB detection, treatment and notification. However, integration still remains a challenge owing to factors such as the low index of suspicion for TB, complexity in screening children for TB, unequal incentives among HCWs, questionable ownership of the intervention, negative HCW attitudes, sociocultural misconceptions as well as the COVID-19 pandemic. Efforts to facilitate this integration must seek to align the systematic screening for TB program with HCWs training and work schedules, promote continuous performance management as well as leverage HCWs collaboration within the primary healthcare facilities.

Finding the missing TB cases will require contextual adaptation of the systematic screening for TB program and strengthening the health system. Further research to quantify the extent of integration of systematic screening for TB in each of the health system building blocks is required to design interventions targeted at strengthening specific weak aspects of integration.

## Supplementary Information

**Additional file 1.** COREQ (COnsolidated criteria for REporting Qualitative research) Checklist.

## Data Availability

The datasets used and/or analyzed during the current study are available from the corresponding author on reasonable request.

## Abbreviations

ACF: Active Case Finding
CHWs: Community Health Workers
DOTS: Direct Observed Treatment, Short-course
HBCs: High TB burden countries
HCWs: Healthcare workers
LMIC: Low- and Middle-Income Countries
MoH: Ministry of Health in Zambia
NSP: National Strategic Plan for Tuberculosis Prevention, Care and Control 2017–2021
OPDs: Outpatient Departments
TB: Tuberculosis
WHO: World Health Organization
